# P-814. Perceptions of implementing point of care testing for respiratory pathogens in primary care: a qualitative study

**DOI:** 10.1093/ofid/ofaf695.1022

**Published:** 2026-01-11

**Authors:** Lauren Dutcher, Elynna Volkova, Lee Ang, Jeffrey Millstein, Jeffrey Tokazewski, Anne Jaskowiak, Keith W Hamilton, Ebbing Lautenbach, Peter Cronholm

**Affiliations:** University of Pennsylvania Perelman School of Medicine, Philadelphia, Pennsylvania; University of Pennsylvania Perelman School of Medicine, Philadelphia, Pennsylvania; University of Pennsylvania Perelman School of Medicine, Philadelphia, Pennsylvania; University of Pennsylvania Health System, Philadelphia, Pennsylvania; University of Pennsylvania Health System, Philadelphia, Pennsylvania; University of Pennsylvania, Philadelphia, Pennsylvania; University of Pennsylvania Perelman School of Medicine, Philadelphia, Pennsylvania; University of Pennsylvania, Philadelphia, Pennsylvania; University of Pennsylvania, Philadelphia, Pennsylvania

## Abstract

**Background:**

Unnecessary antibiotic prescribing in primary care (PC) for patients with upper respiratory tract infections (URIs) remains common. Point of care (POC) testing using an expanded panel of respiratory pathogens has been proposed as a tool to improve antibiotic prescribing in PC. We sought to identify PC clinician and staff perceptions about POC testing for a panel of 14 respiratory pathogens, with a focus on antibiotic stewardship.

**Table 1.**
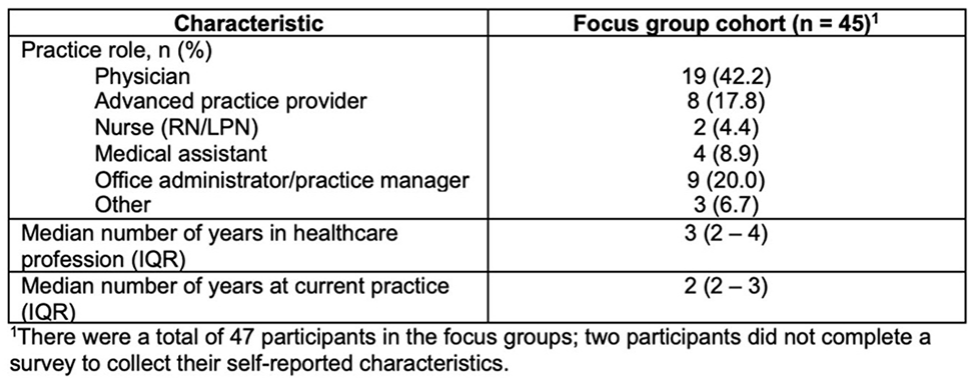
Focus group participant characteristics across five primary care practices

**Table 2.**
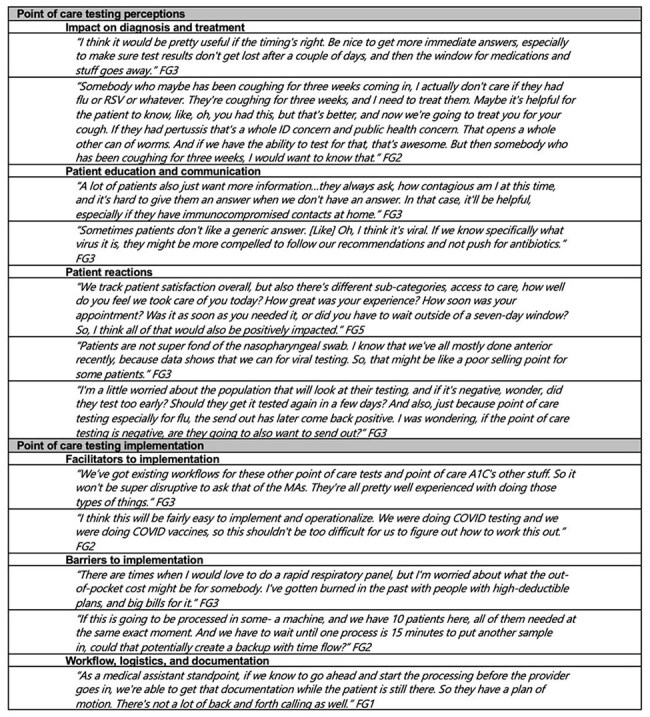
Key themes regarding point of care expanded respiratory pathogen testing identified in primary care focus groups with example quotes

**Methods:**

Five focus groups were conducted between February and March 2025 among five PC practices in Pennsylvania and New Jersey. Focus group participants were comprised of clinicians and practice staff. Topics included perceptions of POC respiratory pathogen testing for patients with URI symptoms, potential workflows, barriers and facilitators to test implementation. Focus groups were audio-recorded, transcribed, cleaned, deidentified, and entered into NVivo 15 software to support coding and analysis. A codebook was developed using an integrated approach including a deductive grounded review of emergent content and deductive coding for a priori implementation domains across transcripts. Two team members double-coded all transcripts (K = 0.93).

**Results:**

A total of 47 respondents participated across 5 practices (n = 7, 14, 11, 10, 5 by practice); (Table 1). The main perceived potential benefits of POC respiratory pathogen testing included decreased antibiotic prescribing and increased patient satisfaction. Positive impacts on community health knowledge, patient education and communication, treatment plans, and administrative labor were also mentioned (Table 2). Barriers to implementation included concerns regarding cost, workflow and time constraints. Prior implementation of similar testing was identified as the main facilitator in testing adoption. Participants’ main concerns included workflow and responsibilities, test documentation, along with test sensitivity and specificity.

**Conclusion:**

Perceptions toward respiratory pathogen POC testing in PC patients with URI symptoms were positive, with an anticipated impact on antibiotic prescribing and patient satisfaction. Multiple logistic factors, barriers and facilitators were identified that could impact the success of implementation.

**Disclosures:**

Keith W. Hamilton, MD, BioMerieux: Grant/Research Support

